# Interobserver agreement of [^68^Ga]Ga-PSMA-11 PET/CT images interpretation in men with newly diagnosed prostate cancer

**DOI:** 10.1186/s13550-020-0596-4

**Published:** 2020-02-28

**Authors:** Céline Derwael, Olivier Lavergne, Pierre Lovinfosse, Vlad Nechifor, Mallory Salvé, David Waltregny, Roland Hustinx, Nadia Withofs

**Affiliations:** 1 Division of Nuclear Medicine and Oncological Imaging, Department of Medical Physics, CHU of Liege, Avenue de l’Hôpital, 1, 4000 Liege, Belgium; 2 Department of Urology, CHR of Liege, Liege, Belgium; 3 GIGA-CRC in vivo imaging, University of Liège, Liege, Belgium; 4 Department of Urology, CHU of Liege, Liege, Belgium

**Keywords:** Interobserver agreement, PSMA-RADS, PROMISE, miTNM, PSMA PET, Standardized evaluation, Interpretation, Criteria

## Abstract

**Background:**

Prostate-specific membrane antigen (PSMA) ligand PET/CT has already provided promising results in prostate cancer (PC) imaging, yet simple and reproductible reporting criteria are still lacking. This study aimed at retrospectively evaluating interobserver agreement of [^68^Ga]Ga-PSMA-11 PET/CT images interpretation according to PC molecular imaging standardized evaluation (PROMISE) criteria and reproducibility of PSMA reporting and data systems (RADS).

**Methods:**

Forty-three patients with newly diagnosed, histologically proven intermediate- or high-risk PC, eligible for radical prostatectomy and who underwent [^68^Ga]Ga-PSMA-11 PET/CT before surgery were retrospectively included. Three nuclear medicine physicians (2 experienced and 1 resident) independently reviewed PET/CT images. Interpretation of [^68^Ga]Ga-PSMA-11 PET/CT images was based on PROMISE criteria including miTNM staging and lesions miPSMA expression score visual estimation and PSMA-RADS version 1.0 for a given scan. Readers’ agreement was measured using Krippendorff’s coefficients

**Results:**

Agreement between observers was almost perfect (coefficient ≥ 0.81) for miM; it was substantial (coefficient ≥ 0.61) for the following criteria: miT, miN, PSMA-RADS, and miPSMA expression score of primary PC lesion and metastases. However, agreement was moderate (coefficient = 0.41–0.60) for miPSMA score of positive lymph nodes and for detection of PC primary lesion.

**Conclusion:**

Visual interpretation of [^68^Ga]Ga-PSMA-11 PET/CT images in patients with newly diagnosed PC in a clinical setting leads to at least substantial agreement for PROMISE criteria and PSMA-RADS classification except for PC primary lesion detection and for miPSMA expression scoring of positive lymph nodes that might have been hampered by the interindividual variability of reference organs PSMA expression.

## Introduction

Prostate-specific membrane antigen (PSMA) ligand positron emission tomography (PET) combined with computed tomography (CT) radically improved prostate cancer (PC) imaging thanks to its superior sensitivity compared to CT and bone scintigraphy [1]. Consequently, PSMA ligand PET/CT is currently recommended for the early detection of recurrence site in patients with PC biochemical recurrence [2]. PSMA ligand PET/CT is currently being investigated in the diagnostic work-up in patients with intermediate- or high-risk localised PC for the detection of lymph nodes and/or metastatic disease that would significantly modify the patient’s therapeutic management [3].

Furthermore, harmonisation of PSMA-ligand PET/CT images interpretation is warranted in order to provide standardised reports not only in clinical trials but also in clinical routine practice [4]. Standardised interpretation criteria have been first proposed by Fanti et al. in 2017 and assessed using the Delphi approach of consensus between experts of seven international PET facilities to detect recurrent PC lesions [5]. As it is the case in other areas of imaging that adopt reporting and data systems (RADS) to standardize the interpretation and reporting of findings from a specific imaging modality, Rowe et al. proposed a PSMA-RADS version 1.0 [6]. Later, Eiber et al. proposed a molecular imaging TNM system (miTNM, version 1.0) incorporating PSMA-ligand PET/CT findings into TNM classification [7].

Prior prospective evaluation and validation of these approaches is needed before being able to implement them in clinical trials and routine clinical practice. Authors who proposed PSMA-RADS classification showed an excellent interobserver agreement for an overall scan when applying this classification to imaging interpretation of [^18^F]DCFPyL PET/CT in a population of patients with PC, the majority of whom had already received prior therapy [8, 9]. The inter-reader agreement of the PC molecular imaging standardized evaluation (PROMISE) proposed by Eiber et al. was substantial for interpretation of [^68^Ga]Ga-PSMA-11 PET/CT in a population of patients with biochemically recurrent PC [7, 10], though authors recently showed that PROMISE criteria agreement was significantly lower for [^18^F]fluciclovine than for [^68^Ga]Ga-PSMA-11 [11]. More recently, Toriihara et al. tested the three standardised interpretation criteria proposed by Fanti et al., Rowe et al. and Eiber et al. in a population of patients who underwent either [^68^Ga]Ga-PSMA-11 PET/MR for PC primary staging or [^68^Ga]Ga-PSMA-11 PET/CT for recurrent PC [12]. They revealed at least substantial agreement of the three classification systems, except in the evaluation of distant metastases based on PSMA-RADS [12].

The aim of the present study was to evaluate interobserver agreement of [^68^Ga]Ga-PSMA-11 PET/CT images interpretation according to PROMISE criteria and PSMA-RADS classification in a population of patients with PC in a preoperative setting [6, 7].

## Materials and methods

### Patients

Patients with PC who underwent preoperative ^68^Ga-PSMA-11 PET/CT from September 2017 to March 2019 were retrospectively consecutively included with approval of the local ethics committee (EudraCT number 2019-002269-36).

Inclusion criteria were histologically proven intermediate- or high-risk PC according to D’Amico classification system, no prior PC treatment and eligible for radical prostatectomy [13]. To evaluate the risk of lymph node involvement, Briganti’s score was calculated for each patient [14].

### [^68^Ga]Ga-PSMA-11 PET/CT

[^68^Ga]Ga-PSMA-11 radiolabelling method is detailed in supplementary material [15]. A mean activity of [^68^Ga]Ga-PSMA-11 of 154 MBq (range, 124–170 MBq) was injected intravenously. Whole-body images from vertex to upper thigh with both arms elevated above the head if possible were acquired after a median interval of 64 min (range, 44–91 min) post-injection in a GEMINI TF Big Bore or a GEMINI TF 16 (Philips Medical Systems, Cleveland, OH, USA). A very low-dose CT (3-mm slice thickness; tube voltage 120 kV and tube current-time product 25 mAs) was performed for attenuation correction, followed by a PET emission scan of 60 to 120 s per bed position depending on the patient’s body mass index (bed overlap of 50%).

Lastly, a CT of the chest, abdomen and pelvis (1-mm slice thickness; tube voltage 120 kV and tube current-time product 150 to 250 mAs depending on the patient’s body mass index) was performed without injection of intravenous contrast agent. All patients received diluted oral contrast (3 g of Telebrix). PET images were reconstructed with standard 4 × 4 × 4 mm^3^ voxels using iterative list mode time-of-flight algorithm, and corrections for attenuation, dead-time, random and scatter events were applied.

### Prostate cancer lesion definition

One nuclear medicine physician resident with 2-year experience and two experienced nuclear medicine physicians (both with 4-year experience in interpreting PSMA PET/CT and 9-year and 14-year experience in PET/CT imaging, respectively) independently reviewed [^68^Ga]Ga-PSMA-11 PET/CT images blinded to clinical data and postoperative pathologic outcomes.

Within the prostate gland, a focal area of increased [^68^Ga]Ga-PSMA-11 uptake higher than surrounding prostatic background was considered suggestive of a PC primary lesion.

A lymph node metastasis was defined as a lymph node with suspicious focal increased [^68^Ga]Ga-PSMA-11 uptake higher than surrounding background independent of the short-axis diameter or a lymph node with no [^68^Ga]Ga-PSMA-11 uptake but with a short axis > 8 mm in the pelvis and > 10 mm outside the pelvis [2, 16].

Prostate cancer bone metastasis was defined as either a very high bone focal uptake of [^68^Ga]Ga-PSMA-11 independent of underlying bone abnormality in CT image or a bone suspicious CT lesion, osteolytic or sclerotic, with no or mild [^68^Ga]Ga-PSMA-11 uptake. Other pathologic findings suggestive of PC lesions detected in CT images and with no [^68^Ga]Ga-PSMA-11 uptake were also reported.

Equivocal findings were avoided as much as possible, and classification into malignant or benign lesion was left to the discretion of the observer. Benign findings were not described.

### miTNM

See Additional file 1: Table S1.

#### Primary tumor (miT)

The number of focal prostate primary lesions and the location within the prostate gland, left lobe and/or right lobe or median location were described. Additionally, the presence or not of a mild diffuse prostate gland [^68^Ga]Ga-PSMA-11 uptake was specified.

The invasion by a primary prostate lesion to seminal vesicles or other adjacent organs was reported. Local miT staging was based on the extent and organ confinement: miT0 in the absence of visible primary prostate lesion, miT2 for organ-confined detected primary prostate lesion with miT2u for unifocal lesion and miT2m in the presence of multiple prostate lesions, miT3b when one or both seminal vesicle invasion was suspected and miT4 for tumours invading adjacent structures other than seminal vesicles [7]. The miT1 category was not used to avoid confusion with the clinicopathologic TNM classification in which T1 defines a tumour too small to have correlation on palpation or any type of imaging [7].

#### Pelvic lymph nodes (miN)

N staging was classified as described by Eiber et al. [7]. The location and number of positive lymph nodes were specified. The short and long axes of lymph nodes of minimum 2-mm axis were measured. The nodal involvement was categorised as miN1a if a single pelvic nodal region was involved or miN1b if multiple nodal regions were involved.

#### Extra-pelvic lymph nodes and distant metastases (miM)

In accordance with the clinicopathologic TNM classification, the involvement of extra-pelvic lymph nodes was considered miM1a; location of positive lymph nodes was reported according to Eiber et al. standard template [7].

Stage was miM1b in the presence of bone metastasis, and the pattern of bone involvement was classified as unifocal, oligometastatic (*n* ≤ 3 metastasis), disseminated or diffuse [7]. Stage was miM1c if other organs were involved.

### miPSMA score

Using the inverted grey scale PET images, a visual estimation of [^68^Ga]Ga-PSMA-11 uptake, the miPSMA score, was estimated for every detected positive lesion in the prostate gland, lymph nodes and metastases, according to Eiber et al. miPSMA scoring system [7]. The miPSMA score was defined as follows: score 0 when lesion uptake was below blood pool, score 1 when uptake was equal to or above blood pool and lower than the liver, score 2 when uptake was equal to or above liver and lower than parotid gland and score 3 for lesion with uptake equal to or above parotid gland. Score was reported as 0, 1, 2 or 3 for no, low, intermediate or high PSMA expression, respectively.

In the case of lymph nodes, the correlation between the PSMA expression score and the size of lymph nodes was tested.

In order to visually differentiate the uptake of the liver and parotids, the upper standardised uptake value (SUV) window threshold was adapted until the liver uptake and parotid uptake could be distinguished visually. The upper scale SUV value set before the visual estimation of the miPSMA score was reported for each patient. The SUV_max_ and SUV_mean_ of the liver and parotids were also estimated by drawing a spherical volume of interest of 3-cm diameter in the liver and of 1.5 cm in the parotid.

### PSMA-RADS version 1.0 classification

Additionally, PET/CT scans were classified at the patient’s level according to the PSMA-RADS version 1.0 classification including five categories described by Rowe et al. [6]*.* PSMA-RADS version 1.0 classification is presented in Additional file 1: Table S2. This classification does not apply to the primary tumour, and therefore, in the presence of the primary PC only (miN0M0), the scan was classified PSMA-RADS-not applicable (NA) excluding the primary PC.

### Statistics

The percentage of agreement between the three observers was assessed, and the interobserver variability was measured by Cohen’s kappa and Krippendorff’s alpha coefficients (K’s alpha) [17]. Values of kappa and alpha statistics ranged from − 1 to 1, and guideline for interpreting the degree of agreement was as follows: total disagreement ≤ 0.01, slight agreement = 0.01–0.20, fair agreement = 0.21–0.40, moderate agreement = 0.41–0.60, substantial agreement = 0.61–0.80, and almost perfect agreement = 0.81–1.00. The agreement was measured for every PROMISE criterion, including miTNM classification and miPSMA score, and for miRADS classification. The kappa and K’s alpha coefficients 95% confidence interval (95% CI) were calculated using bootstrap method.

The intraclass correlation coefficient (ICC) of the number of PC primary lesions between observers was tested using ANOVA-2.

All lymph nodes short and long axes were measured, and Spearman’s correlation between lymph node size and miPSMA expression score was calculated.

Results were considered statistically significant when *P* value was 0.05 or less.

## Results

Forty-three patients were included. Patient characteristics are presented in Table 1.

**Table 1:** Patient characteristics

Characteristic (*n* = 43 patients)	Value*
Median age, range	65 (47–79) years
Median weight, range	87 (63–120) kg
Median PSA level, range at imaging	10.3 (2.62–110) ng/mL
Risk group classification based on D’Amico’s classification system [13]	
Number of patients at intermediate risk	14 (32.5%)
Number of patients at high risk	29 (67.5%)
Number of patients with > 5% risk of lymph node involvement according to Briganti et al. [14]	37 (86%)
Gleason score**	
6	2 (4.6%)
7–8	33 (76.8%)
9–10	8 (18.6%)
ISUP grade (2014 ISUP grading system)	
1	2 (4.6%)
2	14 (32.6%)
3	8 (18.6%)
4	5 (11.6 %)
5	14 (32.6 %)
Clinical T stage	
cT1-2a	27 (62.8%)
cT2b	7 (16.3%)
cT2c	4 (9.3%)
cT3-4	5 (11.6%)

*ISUP* = International Society of Urological Pathology

*Values are reported as numbers of patients, with percentages of patients in brackets, unless otherwise indicated

**Biopsy Gleason score for 16 patients and surgery Gleason score for 27 patients

### miTNM

**Fig. 1:**
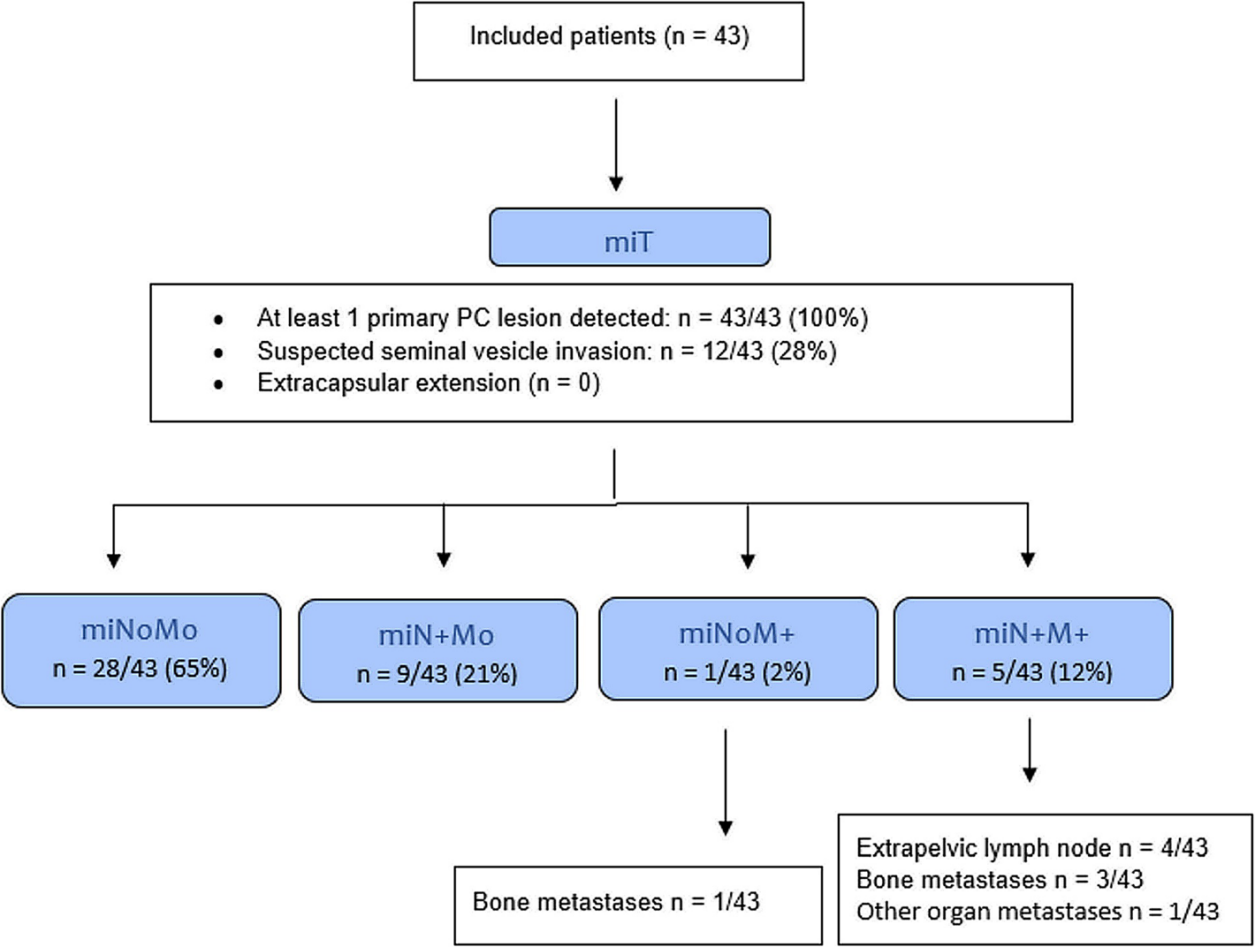
Flowchart of miTNM classification of patients

The miTNM classification of patients is presented in Fig. 1. Interobserver agreement on the visual interpretation of primary tumour, lymph nodes, extra-pelvic lymph nodes and distant metastases are resumed in Table 2.

**Table 2:** Observers (*n* = 3) agreement for miTNM and PSMA-RADS classification

	Agreement K’s alpha (95% confidence interval)	Concordance among observers: *n* pts (%)
miTNM	0.64 (0.48–0.76)	26/43 (60%)
miT	0.64 (0.46–0.78)	29/43 (67%)
miN	0.76 (0.56–0.91)	37/43 (86%)
miM	0.94 (0.81–1.00)	42/43 (98%)
PSMA-RADS	0.76 (0.56–0.90)	36/43 (84%)

The miTNM classification was concordant for 26/43 (60%) patients, and observers’ agreement was substantial (K’s alpha 0.64; 95% CI 0.48–0.76).

#### Primary tumor (miT)

At least one focal anomalous uptake of [^68^Ga]Ga-PSMA-11 was detected in the prostate of all patients. The number of focal lesions in the prostate gland was concordant in 26/43 patients (60%; *n* = 1 focal prostate lesion in 20/43 patients and *n* = 2 focal lesions in 6/43 patients), and interobserver agreement was moderate (K’s alpha 0.50; 95% CI 0.34–0.66). 43/43 (49%) patients who had 1 focal uptake only, observers were discordant for 1/43 (2%) patient only, for which one observer did not describe any significant focal uptake; whereas multiple focal prostate uptake was described by at least one observer in 22/43 (51%) patients and observers were discordant regarding the number of lesions for 16/22 (72%) patients.

The number of prostate focal lesions was statistically significantly different between the three observers overall (observer #1: *n* = 64; observer #2: *n* = 56; and observer #3: *n* = 78; ANOVA-2 *P* < 0.0001) and in the two-by-two comparison (ANOVA-2 *P* = 0.0019; *P* = 0.0002; and *P* = 0.031).

The presence or absence of a diffuse [^68^Ga]Ga-PSMA-11 uptake in the prostate gland was concordant in 26/43 (60%) patients, and the agreement was fair (K’s alpha 0.25; 95% CI 0.01–0.46). Interestingly, prostate gland diffuse uptake was more frequently described by observer #2 who detected a lower number of focal lesions (*n* = 18 patients, versus *n* = 8 and *n* = 3 for other observers).

There was an agreement for the seminal vesicle invasion or not in 36/43 (84%) patients, and the interobserver agreement was substantial (K’s alpha 0.64; 95% CI 0.35–0.86). No extracapsular extension was observed.

The miT classification was concordant for 29/43 (67%) patients with substantial agreement (K’s alpha 0.64; 95% CI 0.46–0.78).

#### Pelvic nodes (miN)

Positive lymph nodes were detected in 14/43 (33%) patients. Positive lymph nodes were located in the following regions: internal iliac (*n* = 10/43; 23%), external iliac (*n* = 10/43; 23%), obturator (*n* = 2/43; 5%), common iliac (*n* = 5/43; 12%), pararectal (*n* = 4/43; 9%) and presacral (*n* = 3/43; 7%). A total of 66 positive lymph nodes was detected by observers, and the median number of positive lymph node per patient was 3 (range, 1–15). The number of positive lymph nodes detected by observers was not statistically different (ICC 0.96 with 95% CI 0.95–0.98; ANOVA-2 *P* = 0.36).

The miN classification was concordant for 37/43 (86%) patients: *n* = 29/43 (67%) N0 and *n* = 8/43 (19%) N1b. The miN classification was discordant for 6/43 (14%) patients: *n* = 3/43 (7%) N0 versus N1a; *n* = 2/43 (5%) N1a versus N1b; and *n* = 1/43 (2%) N1a versus N1b versus N0. Nevertheless, the agreement was substantial (K’s alpha 0.76; 95% CI 0.56–0.91).

The median (range) short axis of positive lymph nodes was 7.0 (2.3–15.7) mm, and the median (range) long axis was 9.7 (3.4–28.9) mm. Positive lymph node short axis was non-measurable (< 2 mm) for 5/66 (7.6%), ≥ 2 mm and < 8 mm for 38/66 (57.6%) and ≥ 8 mm for 23/66 (34.8%).

#### Extra-pelvic nodes and distant metastases (miM)

Positive extrapelvic retroperitoneal lymph nodes were detected in 4/43 (9%) patients. In one patient, a metastasis was detected in the right vas deferens. Bone metastases were detected in 4/43 (9%) patients and were located in the pelvis in all patients, in the spine in 3/4 patients, in the ribs in 3/4 patients and in other locations (sternum or clavicle and scapula) in 2/4 patients. Both positive extrapelvic retroperitoneal lymph nodes and bone metastases were present in 2/43 (5%) patients.

**Fig. 2:**
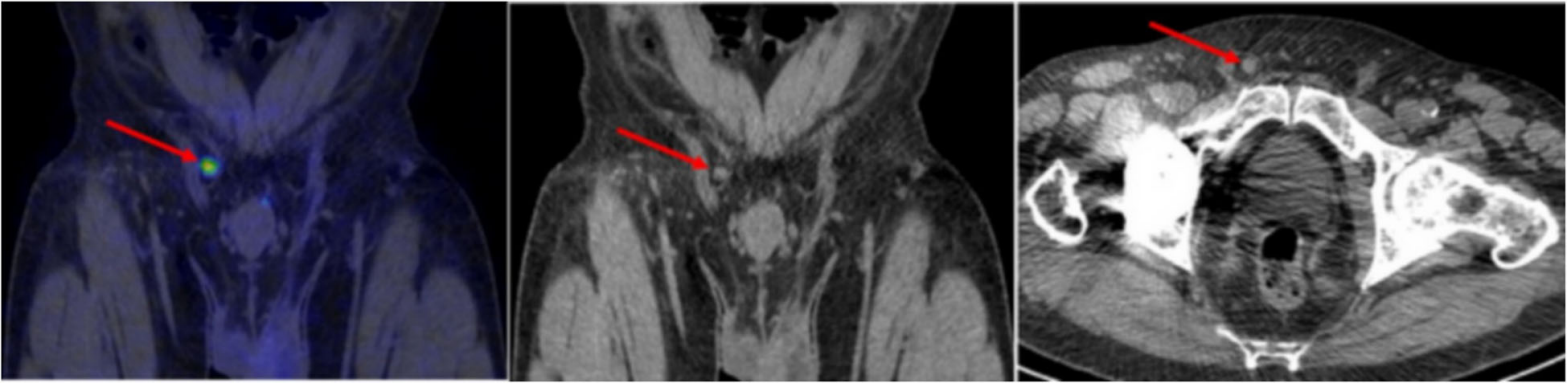
The only case of discordant miM classification was a case of a patient with a vas deferens lesion considered as visceral metastasis by two observers and as inguinal lymph node for the other one

The miM classification was concordant for 42/43 (98%) patients with almost perfect agreement (K’s alpha 0.94; 95% CI 0.81–1.00) (Fig. 2).

### miPSMA score

Considering the primary PC miPSMA expression score, the observers’ agreement was substantial (Table 3); miPSMA score was concordant in 34/43 (79%) patients: miPSMA score 3 (26/34; 76.5%), miPSMA score 2 (7/34; 20.5%) and miPSMA score 1 (1/34; 3%). The miPSMA score of the primary PC was discordant in 9/43 (21%) patients: score 3 versus 2 in 7/9 (78%) patients and score 1 versus 2 in 2/9 (22%) patients.

**Table 3:** miPSMA score observers’ agreement K’s alpha (95% confidence interval)

Lesion category	miPSMA 3-point score: 1, 2 or 3	miPSMA 2-point score: 1–2 versus 3	miPSMA 2-point score: 1 versus 2–3
Primary prostate lesion^†^	0.69 (0.51–0.85)	0.75 (0.55–0.90)	††
Lymph nodes	0.59 (0.50–0.68)	0.65 (0.55–0.75)	0.67 (0.58–0.77)
Metastases	0.68 (0.58–0.77)	0.76 (0.66–0.85)	0.76 (0.66–0.85)

[^68^Ga]Ga-PSMA-11 uptake miPSMA score 1: uptake ≥ blood pool and < liver; score 2: uptake ≥ liver and < parotids; score 3: uptake ≥ parotid gland

†Score of the most intense focal primary prostate lesion

††Not calculated as only one miPSMA score 1 described by the observers

Considering miPSMA expression score of positive lymph nodes, observers’ agreement was moderate (Table 3); out of the 46/66 (70%) positive lymph nodes detected by all observers, miPSMA score was concordant in 27/46 (59%): miPSMA score 3 (24/27; 89%), miPSMA score 2 (1/27; 4%) and miPSMA score 1 (2/27; 7%). The miPSMA score was discordant in 19/46 (41%): score 3 versus 2 in 12/19 (63%) lymph nodes, score 1 versus 2 in 6/19 (32%) lymph nodes and score 1 versus 3 in 1/19 (5%) lymph nodes.

A statistically significant correlation was found between miPSMA expression score and lymph nodes size, for both short and long axes (Spearman correlation coefficients are presented in Additional file 1: Table S3).

The observers’ agreement for the miPSMA score of metastases was substantial (Table 3). The miPSMA score was concordant in 24/40 (60%) metastases: score 3 for 23/24 (96%) and score 2 for 1/24 (4%) metastases, whereas it was discordant in 16/40 (40%) metastases (10/28 bone metastases and 6/12 positive extra-pelvic lymph nodes): score 2 versus 3 for 8/16 (50%) metastases, score 1 versus score 2 for 6/16 (37.5%) and score 1 versus 2 versus 3 for 2/16 (12.5%) metastases.

The median (range) SUV_max_ and SUV_mean_ in the parotids were 16.7 (7.2–32.8) and 12.7 (6.2–24.9), respectively. The median (range) SUV_max_ and SUV_mean_ in the liver were 7 (6–14.7) and 4.7 (3.8–12), respectively. Using the inverted grey scale PET images, the median (range) upper SUV window threshold set to visually differentiate parotids and liver activities in order to estimate the miPSMA score was 6 (5–12). There was a significant correlation between the upper SUV window threshold value and both liver SUV_mean_ and SUV_max_ (*P* < 0.0001) while there was no significant correlation with parotids SUVs (*P* > 0.05).

### PSMA-RADS version 1.0 classification

The observers’ agreement for PSMA-RADS classification was substantial (K’s alpha 0.76, 95% CI 0.56–0.90). The PSMA-RADS classification was concordant in 36/43 (84%) patients: PSMA-RADS-NA in 25/36 (69%) patients and PSMA-RADS-5 in 11/36 (31%) patients. The PSMA-RADS classification was discordant in 7/43 (16%): PSMA-RADS-NA versus PSMA-RADS-5 (*n* = 4/7) or PSMA-RADS-NA versus PSMA-RADS-1B or PSMA-RADS-3B or PSMA-RADS-4 (*n* = 3/7).

Out of the 28/43 (65%) patients with miN0M0, the PSMA-RADS was not applied in 25/28 (89%) and applied in 3/28 (11%): PSMA-RADS-1B (bone focal uptake in femur considered definitely benign), PSMA-RADS-3B (bone focal uptake in 5th lumbar vertebra considered equivocal) or PSMA-RADS-4 (bone focal uptake in 12th dorsal vertebra with lack of anatomical abnormality).

Lastly, Cohen’s kappa coefficients were similar to K’s alpha coefficients for all analyses.

## Discussion

Development and validation of standardized imaging interpretation criteria is essential both for the harmonisation of acquired data in clinical trials enabling results comparability and eventually to allow better communication with referring clinicians. This work showed that the use of PROMISE criteria and PSMA-RADS version 1.0 classification for the visual interpretation of [^68^Ga]Ga-PSMA-11 PET/CT images in a clinical setting leads to substantial agreement for miTNM, miT and miN staging and PSMA-RADS classification [6, 7]. This study assessed these criteria in a homogeneous population of patients with newly diagnosed PC eligible for surgery. Previous studies also showed [^68^Ga]Ga-PSMA-11 PET/CT image interpretation substantial agreement in patients with recurrent PC using PROMISE criteria or criteria based on Delphi approach of consensus between experts and in patients with newly diagnosed PC but with non-standardised criteria [5, 10, 18, 19]. Additionally, we demonstrated inter-reader substantial agreement with a less experienced physician resident with 2-year experience [20]. Our results are in line with Toriihara et al. who showed at least substantial agreement of PROMISE and PSMA-RADS criteria in a group of patients (*n* = 47) who underwent [^68^Ga]Ga-PSMA-11 PET combined with magnetic resonance imaging for initial staging; a point-by-point comparison of the results of Toriihara et al. and ours is presented in Additional file 1: Table S4 [12].

Agreement for the detection of extra-pelvic nodes and distant metastases (miM) was almost perfect; the agreement for the presence or not of metastases was concordant for all patients (37/43 M0 and 6/43 M1a/1b). This result is in line with a previous study, and accurate interpretation of the presence or not of distant metastases is of particular importance as it has significant impact on treatment decision, ruling out surgical option [12, 19].

**Fig. 3:**
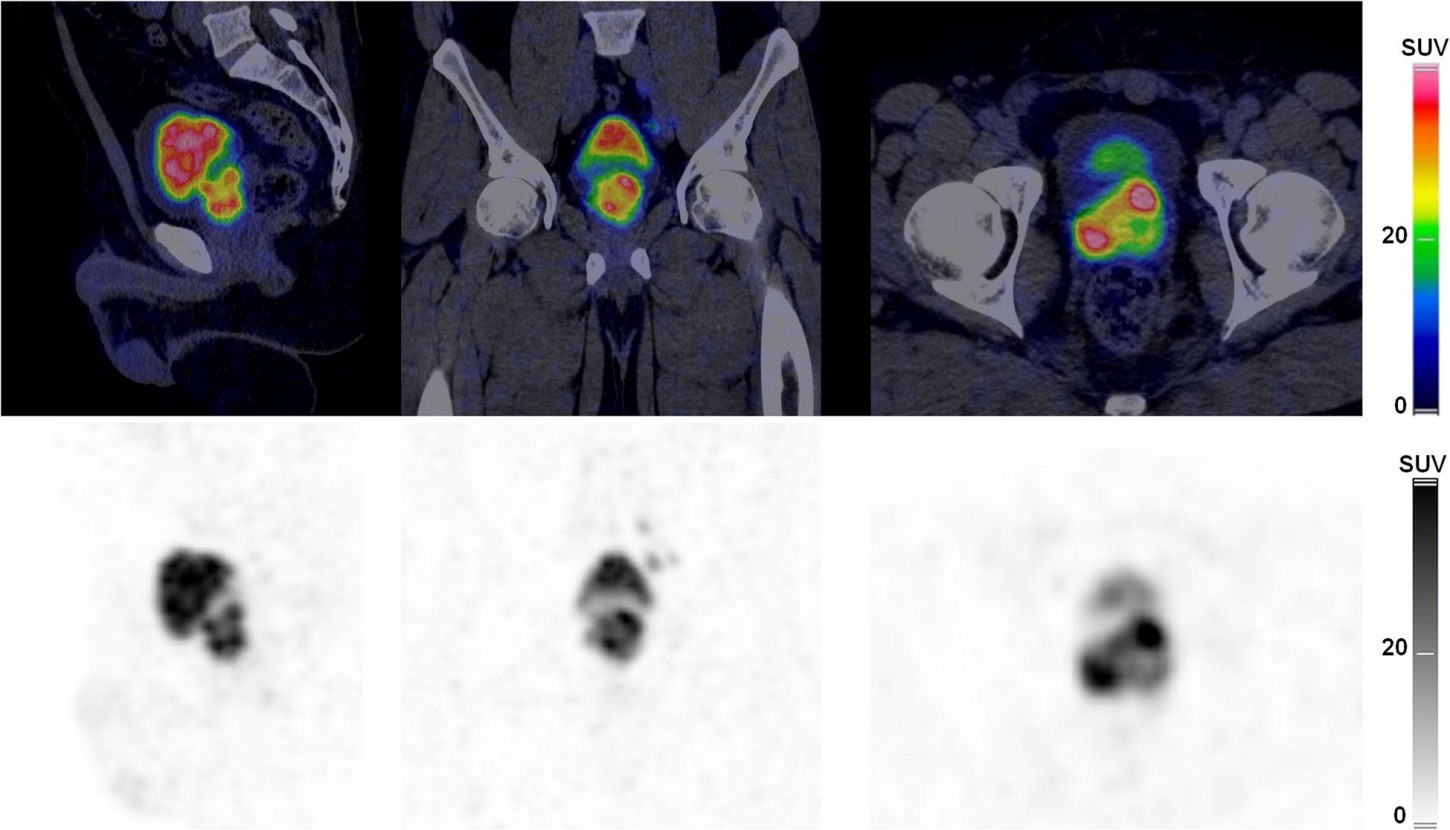
The PET/CT images show a case of observers agreement on diffuse [^68^Ga]Ga-PSMA-11 uptake in the prostate gland but discordance of the number of prostate primary lesions: one left focus and one right focus were described by observers #1 and #2 while two left foci and three right foci were detected by observer #3

On the other hand, the agreement was moderate for the detection of PC primary lesion. One of the observers described prostate gland diffuse mild [^68^Ga]Ga-PSMA-11 uptake more frequently than the two other observers while one observer detected a greater number of prostate gland focal lesions (Fig. 3). This might be related to the absence of detailed and validated standardized criteria for the definition of a positive prostatic primary lesion based on [^68^Ga]Ga-PSMA-11 PET/CT: PROMISE criteria focus more on the extent and organ confinement of the primary prostate lesion and do not specifically define a prostatic primary lesion; PSMA-RADS is not applicable on the primary prostate cancer, and Fanti et al. criteria were developed to detect prostate cancer recurrent lesions [5–7]. However, qualitative examination is usually based on the detection of focal [^68^Ga]Ga-PSMA-11 uptake higher than prostatic surrounding background [5, 19, 21–23]. Disagreement in the interpretation of prostatic lesions was also pointed out by Toriihara et al. and might have been due to differences in interpretation of moderate foci or diffuse uptake [12]. Therefore, criteria should be further refined in order to describe clinically important PC as it has been demonstrated with multiparametric magnetic resonance imaging of the prostate [24]. One suggestion would be to describe focal intense prostate lesions with visually higher uptake than the liver (similar or greater than parotid activity: miPSMA score 3) as PSMA expression has been proven to be higher in more aggressive PC as defined by Gleason score, with clinically significant cancer defined as Gleason score ≥ 4 + 3 [21, 25, 26]. However, this will have to be prospectively validated by pathology analyses. Describing mild or moderate focal prostatic [^68^Ga]Ga-PSMA-11 uptake might be irrelevant if there is already a prostatic lesion with high intense uptake. For the latter, we showed that the agreement for the miPSMA expression scoring of the most intense focal primary prostate lesion was substantial (Table 3).

**Fig. 4:**
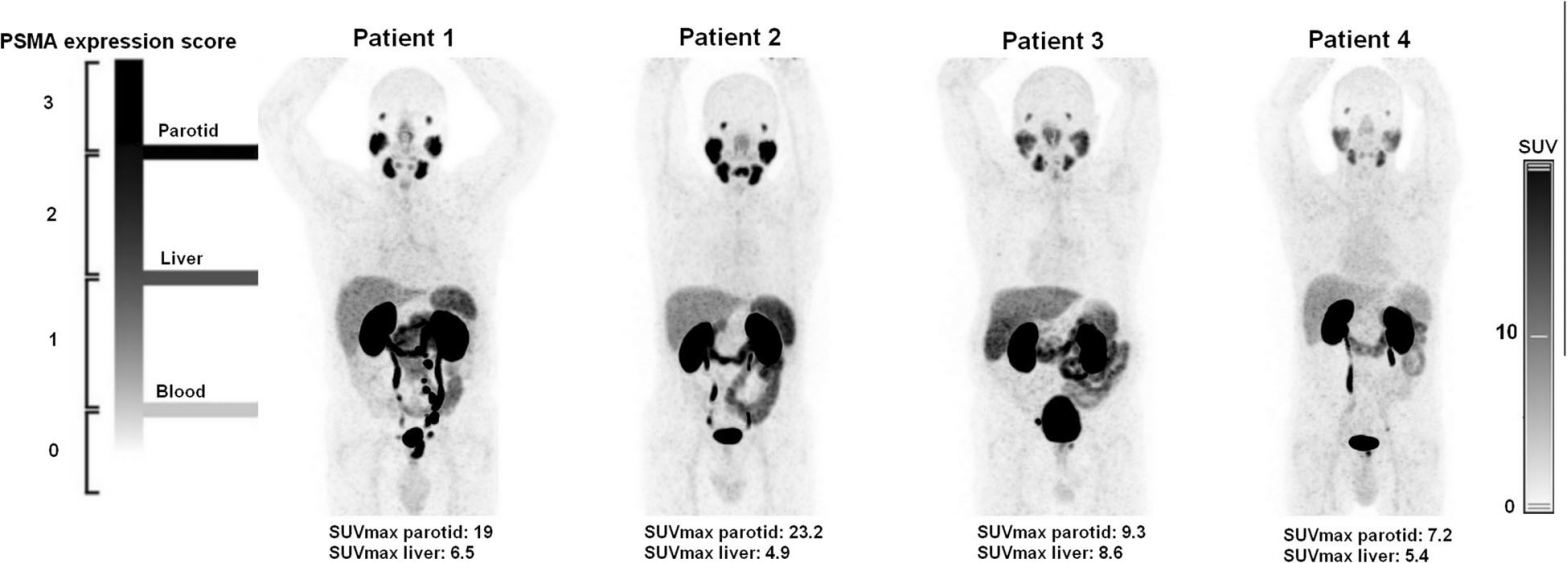
[^68^Ga]Ga-PSMA-11 PET maximum intensity projection images of four patients showing interindividual variability of PSMA expression of the liver and parotid, reference organs. The visual distinction between parotids and liver miPSMA expression scores is simple for patients 1 and 2 while miPSMA expression scores of liver and parotids is visually similar for patients 3 and 4. Left side of the figure adapted from Eiber et al. figure [7]

The agreement of miPSMA expression scoring of positive lymph nodes was moderate between the three observers, but it was substantial between the two experienced nuclear medicine physicians. This is the only analysis where we observed a relation between the concordance of the results and the reader’s experience. Nevertheless, the visual differentiation between parotids and liver activity might be affected by subjectivity regardless of experience (Fig. 4). Furthermore, we pointed out that the upper SUV window threshold had to be manually modified in order to visually differentiate liver and parotid activities for more than half of patients (25/43; 58%), which is confirmed by quantitative analyses showing a wide range of parotids SUV_max_ (7.2–32.8) and liver SUV_max_ (4.8–14.7). When grouping the miPSMA scores in two categories, scores 1–2 versus 3 or 1 versus 2–3, the concordance became substantial. The visual miPSMA scoring proposed by Eiber et al. might be simplified into a binary scoring considering only one reference organ. One further refinement in the interpretation of [^68^Ga]Ga-PSMA-11 PET/CT images is the integration of quantitative analysis to visual analysis. Recently, Gafita et al. introduced a semi-automatic software to assist physicians to quantify tumour burden in cases of patients with metastatic PC [27]. In a future work, the added value of PSMA-ligand positive tumor volume (PSMA-TV), PSMA-ligand positive total lesion (PSMA-TL), PSMA-SUV_mean_ and PSMA-SUV_max_ as well as radiomics features will be investigated for the evaluation of intra-prostatic primary lesion.

One other important reason for developing reproducible PSMA ligand PET images interpretation criteria is the use of PET images for the planning of PSMA-directed radioligand therapy with ^177^Lu-PSMA ligands, for which there is no agreement on what should be considered an “adequate” uptake of PSMA-ligand PET agents [28]. For example, one phase II trial on ^177^Lu-PSMA-617 required a baseline [^68^Ga]Ga-PSMA-11 SUV_max_ at dominant sites of tumour involvement to be at least 1.5 times the SUV_mean_ of the liver [29].

Finally, and most importantly, there were no instances where disagreements among observers would have led to a change in therapeutic management.

One limitation of this study is that readers were not trained with preliminary data sets; PET/CT images interpretation was done in a clinical setting, based on methods well detailed in published articles [6, 7]. The small sample size and the limited number of observers might be additional limitations, and finally, no consensus reading was performed in this work as no confrontation to pathology was made.

## Conclusion

The visual interpretation of [^68^Ga]Ga-PSMA-11 PET/CT images in patients with newly diagnosed PC in a clinical setting leads to substantial agreement for miTNM, miT and miN staging according to PROMISE criteria and PSMA-RADS version 1.0 classification and almost perfect agreement for miM [6, 7]. However, the agreement was moderate for the detection of PC primary lesion and for the evaluation of miPSMA expression scoring of positive lymph nodes.

## Supplementary information

**Additional file 1: Table S1.** miTNM classification adapted from Eiber et al. [7]. **Table S2.** The PSMA-RADS version 1.0 classification schema, adapted from Rowe et al. [6]. **Table S3.** Correlation between positive lymph nodes size and miPSMA score. **Table S4.** Comparison between the present work and Toriihara et al. work [12].

## Data Availability

Please contact author for data requests.
