# Supplementary material for: Interobserver agreement of [68Ga]Ga-PSMA-11 PET/CT images interpretation in men with newly diagnosed prostate cancer
Source: EJNMMI Res. 2020 Feb 28;10:15. doi: 10.1186/s13550-020-0596-4 (PMC7048889; doi:10.1186/s13550-020-0596-4)
Supplement: Supplementary file 1 — Additional file 1: Table S1. miTNM classification adapted from Eiber et al. [7]. Table S2. The PSMA-RADS version 1.0 classification schema, adapted from Rowe et al. [6]. Table S3. Correlation between positive lymph nodes size and miPSMA score. Table S4. Comparison between the present work and Toriihara et al. work [12]. [file 13550_2020_596_MOESM1_ESM.docx]

**TITLE**

Interobserver Agreement of [^68^Ga]Ga-PSMA-11 PET/CT images interpretation

**AUTHORS**

Céline Derwael^1^, Olivier Lavergne^2^, Pierre Lovinfosse^1,3^, Vlad Nechifor^4^, Mallory Salvé^1,3^, David Waltregny^4^, Roland Hustinx^1,3^, Nadia Withofs^1,3^

**Affiliations**

^1^ Division of Nuclear Medicine and Oncological Imaging, Department of Medical Physics, CHU of Liege, Liege, Belgium

^2^ Department of Urology, CHR of Liege, Liege, Belgium

^3^ GIGA-CRC in vivo imaging, University of Liège, Belgium

^4^ Department of Urology, CHU of Liege, Liege, Belgium

**Corresponding author:** nwithofs@chuliege.be

**SUPPLEMENTARY MATERIAL**

**SUPPLEMENTARY METHOD**

**[^68^Ga]Ga-PSMA-11 radiolabelling**

The Germanium-68/Gallium-68 generators were an IGG-100 from Eckert-Ziegler and an Isotope Technologies Garching (ITG) Gallium-68 generator. The radiolabeling automatization conducted on a GAIA synthesizer from Elysia-Raytest, with kits and reagents from ABX. The automation sequence (15 minutes) is a fractionation and SCX cartridge-based post-processing (Gallium-68 purification on a 100 mg Bond Elut SCX column from Agilent) associated with a 4-minute radiolabeling part in 2.340 mL 0.1 M pH 4.0 sodium acetate buffer, plus radical scavengers 5 mg sodium ascorbate, at 90°C [[29](#_ENREF_29)]. This method combines the strength from two different post-processing, an optimized [^68^Ga]Ga-labelling, a final product purification on C-18 column from Waters, a formulation with isotonic NaCl 0.9% and ethanol, a final filtration on a 0.22 µm filter, and an automatic filter integrity test (bubble test point) to insure a robust production.

**SUPPLEMENTARY TABLES**

**Supplementary Table S1** miTNM classification adapted from Eiber et al. [[7](#_ENREF_7)]

| Class | Description |
| --- | --- |
| Local Tumour (miT) |  |
| miT0 | No local tumour |
| miT2 | Organ-confined tumour |
| miT2u | Unifocality |
| miT2m | Multifocality |
| miT3 | Non–organ-confined tumour |
| miT3a | Extracapsular extension |
| miT3b | Tumour invading seminal vesicles |
| miT4 | Tumour invading adjacent structures other than seminal vesicles, such as external sphincter, rectum, bladder, levator muscles, or pelvic wall |
| Regional nodes (miN) |  |
| miN0 | No positive regional lymph nodes |
| miN1a | Single lymph node region harboring lymph node metastases; report location by standardized template [[7](#_ENREF_7)] |
| miN1b | Multiple (≥ 2) lymph node regions harboring lymph node metastases; report locations by standardized template [[7](#_ENREF_7)] |
| Distant metastases (miM) |  |
| miM0 | No distant metastasis Distant |
| miM1 | Distant metastasis |
| miM1a | Extrapelvic lymph nodes; additionally, report location by standardized miM1a template [[7](#_ENREF_7)] |
| miM1b | Bones; additionally, report pattern and involved bones if unifocal or oligometastatic |
| miM1c | Other sites; additionally, report involved organ |

**Supplementary Table S2** The PSMA-RADS version 1.0 classification schema, adapted from Rowe *et al.* [[6](#_ENREF_6)]

| Category | Findings |
| --- | --- |
| PSMA-RADS-1 | Benign |
| A | Lesions without radiotracer uptake that are definitively benign |
| B | Lesions with radiotracer uptake that are definitively benign |
| PSMA-RADS-2 | Likely benign  Low level radiotracer uptake in bone or soft tissue sites that would be atypical for metastatic PC |
| PSMA-RADS-3 | Equivocal |
| A | Equivocal radiotracer uptake in soft tissue lesions such as lymph nodes in a distribution typical for PC |
| B | Equivocal radiotracer uptake in bone lesions that are not clearly benign |
| C | Lesions that would be atypical for PC but have high levels of uptake and may represent a non-prostate malignancy |
| D | Lesions that are concerning for the presence of PC or a non-prostate malignancy but lack radiotracer uptake |
| PSMA-RADS-4 | PC highly likely  Lesions with high radiotracer uptake that would be typical for PC but lack a definitive anatomic abnormality |
| PSMA-RADS-5 | Definitively cancer  Lesions with high level of radiotracer uptake and corresponding anatomic findings that are indicative of the presence of PC |

**Supplementary Table S3** Correlation between positive lymph nodes size and miPSMA score

| Tested correlation | Observer number | Spearman correlation coefficient | *P* value |
| --- | --- | --- | --- |
| Lymph node miPSMA score versus short-axis | 1  2  3 | 0.73465  0.58362  0.65158 | 0.0028  0.0284  0.0217 |
| Lymph node miPSMA score versus long-axis | 1  2  3 | 0.64786  0.71253  0.43005 | 0.0122  0.0042  0.1629 (NS) |

NS = not significant (*P* > 0.05)

**Supplementary Table S4** Comparison between the present work and Toriihara *et al.* work [[12](#_ENREF_12)]

|  | **Present study** | **Toriihara *et al.* study** | |
| --- | --- | --- | --- |
| **STUDY DESIGN** | Retrospective | Retrospective | |
| **PATIENTS**  Clinical setting  Number of patients | Primary staging  43 | Group 1:  Primary staging  47 | Group 2:  BCR  57 |
| **IMAGING**  Tracer  Imaging modality | [^68^Ga]Ga-PSMA-11  PET/CT | [^68^Ga]Ga-PSMA-11  PET/MRI | [^68^Ga]Ga-PSMA-11  PET/CT |
| Number of reviewers  Experience of observers | 3  2 experienced readers (14 and 9 years) and  1 resident with 2 years of experience | 3*  3 experienced readers (14, 11 and 8 years) | |
| **Interpretation criteria**  PSMA-RADS [[6](#_ENREF_6)]  PROMISE [[7](#_ENREF_7)]  EANM [[5](#_ENREF_5)] | Assessed  Assessed  NA | Assessed  Assessed  Assessed | |
| **Statistical analysis** | K’s alpha coefficient | Gwet’s agreement coefficient | |
| **RESULTS**  **PSMA-RADS**  **inter-observer agreement**  Overall scan  Primary PC  LN metastases  Distant metastases | Substantial  NA  NA  NA | Substantial  Substantial  Almost perfect  Almost perfect | Substantial  Substantial  Substantial  Moderate |
| **PROMISE**  **inter-observer agreement**  miT  miN  miM  miTNM  **miPSMA-score**  Primary PC  LN metastases  Distant metastases | Substantial  Substantial  Almost perfect  Substantial  Substantial  Moderate  Substantial | Substantial  Almost perfect  Almost perfect  Substantial  NA  NA  NA | Substantial  Substantial  Substantial  Substantial  NA  NA  NA |
| **EANM**  **inter-observer agreement**  Primary PC  LN metastases  Distant metastases  Final judgement | NA  NA  NA  NA | Substantial  Almost perfect  Almost perfect  Almost perfect | Substantial  Substantial  Almost perfect  Substantial |
| **Inter-criteria agreement** | NA | Almost perfect | Almost perfect except for PSMA-RADS of distant metastases |
| **Intra-observer agreement** | NA | Substantial to almost perfect for all criteria | Substantial to almost perfect for all criteria, except miTNM and overall scan PSMA-RADS that was moderate for one of the 2 readers |
| **Pathological correlation** | None | None | |

* In order to estimate the intra-observer variability, 2 of the 3 reviewers reevaluated all imaging studies 6 months later in the same manner and blinded to the initial reading.

BCR = biochemical recurrence; NA = Not assessed
